# Human Coronary Artery Remodeling, Beginning and End of the Atherosclerotic Process

**DOI:** 10.1371/journal.pone.0000091

**Published:** 2006-12-20

**Authors:** Paul de Groot, R.W. Veldhuizen

**Affiliations:** 1 Department of Cardiology, Medisch Centrum Haaglanden (MCH) Hospital, The Hague, The Netherlands; 2 Department of Pathology, Medisch Centrum Haaglanden (MCH) Hospital, The Hague, The Netherlands; Baylor College of Medicine, United States of America

## Abstract

**Background, Aims of the Study:**

The objective of the study was to relate the progress of coronary artery remodeling to the earliest stages of the atherosclerotic process. For this purpose, a mathematical model for description of dimensional change of the coronary artery wall and its constituent components was developed and applied.

**Materials and Methods:**

The study used coronary artery samples randomly taken from each of 83 consecutive, unselected postmortems. All samples were routinely fixed and processed to paraffin for the preparation of right-angled, 5-micron sections, routinely stained and mounted for subsequent analysis. Computer assisted image analysis, using 32 systematic random, radial sampling lines, was used for interactive measurements of distance from centre of lumen to points defining intima, media and adventitia thickness along the radial intercept, which were subsequently tabled for analysis of variance, calculations of (group –vessel) means, and related to stage of pathology.

**Results:**

Pre-atherosclerotic changes, before any localised changes in especially intima dimensions, are found, consisting of a process of gradual vascular widening, associated with temporally at least partly dissociated increases in width, which as a fraction of total vessel radius show a phased process. In these, the intima first increases, subsequently remains stable, and finally reduces in width proportionally to the increasing diameter. The media shows a similar initial increase, on average stabilising in the third phase after reaching a plateau value in the second. The adventitia, already increasing in phase 1, continues to increase in phase 2, accelerating in phase 3. The complex process, as found, occurs systematically in all vessels, is distributed circumferentially, and precedes the development of localised lesions of the intima.

**Conclusions:**

The findings suggest the existence of a diffuse complex of changes, consisting of a gradual vascular widening followed by narrowing, with associated mural changes reflecting the atherosclerotic process.

## Introduction

Coronary artery morphology and diameter change as a result of atherosclerosis. This phenomenon has already been described in 1987 [1] and has since been demonstrated repeatedly. Initially coronary artery remodeling was held to consist of enlargement of the lumen diameter as a result of a volume change of the intima (positive remodeling) in contrast to the later descriptions of lumen diameter reduction due to a process of “shrinkage” of the wall of the affected vessel [2].

At first coronary artery remodeling was studied and defined exclusively on the basis of histo-pathological investigation. This was followed by in-vivo investigation using intravascular ultra sound methods (IVUS). Although this answered many questions, many were left unanswered. With the unavailability of artery samples in early stages of pathological change and the relative insensitivity of IVUS, both approaches have shortcomings with respect to the problems at hand. These include: when does coronary artery remodeling commence, what is the relative contribution of each component of the arterial wall (intima, media and adventitia) and especially: what is the sequence of changes within each of these. These questions have been left largely un-addressed.

However, both histopathological and IVUS studies have an additional fundamental shortcoming. They both define atherosclerosis to be a segmental disease and thus measure changes in the affected segment relative to a proximal or distal segment of the vessel under examination which by inference is assumed to be normal and unchanged [3], [4]. However, often this is uncertain and the use of the chosen segments as a reference site questionable.

In addition, direct comparison of measurements of vessels of different diameters is almost impossible as the thickness of the vessel wall and its constituents (intima, media and adventitia) are non-linearly related to the diameter of the normal vessel. Comparisons of changes can thus only be made by using a conversion factor normalising measurements for differences in diameter [5].

Even with these limitations it is generally accepted that remodeling of the vessel wall occurs throughout the constituent layers of those parts of the vessel wall affected by non-circumferential lesions [2], [6]. This even when these lesions are small and consist of atherosclerotic plaques limited to the intima [7]. Changes were thought to be limited in their distribution along the circumference to the affected section [8]. However, previous work by the authors shows that the lumen of coronary arteries at the site of lesions, at the time of analysis of the lesions, has in fact increased in diameter as a result of circumferential increase in mass (“growth”) of the vessel wall. In this process the ratio of the thickness of intima to vessel diameter (lumen radius) has remained constant [5]. We may now raise the question of whether this vascular wall change extends beyond such affected sites and in fact this process of dimensional change actually precedes local processes.

Previous morphological studies of coronary arteries using histological sections were limited by the software available at that time for image analysis. The programs and studies used area measurements of its components, lumen and wall, in right angle sections as a basis. Use of directly calculated ratios of these areas in effect disregards the nature of these non-circumferential changes as a form of “averaging”. It is not inconceivable that much early change has gone undetected as a result of this poor signal to noise approach and that our present perceptions of time of onset of pathology and sequence of events are incorrect. The authors have developed a different approach that uses systematic random radial measurements combined with analysis of the variance of the data set for each location, to overcome the loss of information potentially associated with undue use of averaged values as a principle to overcome these shortcomings and has applied this to a series of coronary artery vessels assumed to show only early forms of pathology if any. The purpose was to redefine the earliest period of change in vessels affected by frank atherosclerosis at a later stage.

## Materials and Methods

### Study population

Samples for study were obtained prospectively at autopsy of an unselected series of patients with cause of death other than coronary artery and/or cardiovascular disease. There were no age limits to inclusion in the study. A total of 83 cases were accepted for and included in the study.

“Medisch Centrum Haaglanden” Ethics Committee agreed on study plan and use of material obtained from routine post mortems.

### Coronary artery sampling, histology

After postmortem (part of) hearts with coronary vessels attached were fixed by submersion in phosphate buffered (0.1 M, pH 7.0) formaldehyde 4% for 48 hours prior to further sampling. Right angle through section samples with a length of 4 mm were taken at 4 cm from the aortic origin of the right sided coronary artery and 2 cm from the bifurcation of the communal segment of the left sided coronary artery.

Sections were processed to paraffin by routine processing, 5 micron sections were stained with Haematoxylin and Eosin for assessment.

### Study groups

All samples were recoded and randomised in order to blind observers to age and gender of patients, type and location of vessel sample and causes of death.

For the purpose of later validation of a parameter for progression of pathology, to be derived from computerised image analysis, based on the measurements acquired, sections were grouped using histopathological criteria, as follows:

N: normal artery: intima only a few cells in width along full circumference.

Vessels with circumferential changes only:

AN: possibly abnormal artery: intima widened circumferentially, thickness of intima less than media.

A: abnormal artery: intima widened circumferentially, thickness of intima exceeds that of media.

Vessels with non-circumferential changes:

AE: abnormal artery: intima widened only locally i.e. non-circumferentially, the remainder of the intima cirmference is normal in width.

EN: abnormal artery: extensive, well developed plaque, the remaining circumference is normal in appearance.

EA: abnormal artery: extensive, well developed plaque, the remaining circumference shows pathological changes.

EE: abnormal artery: circumferential atherosclerotic plaque.

Cases with frank non-circumferential plaque lesions are described as group E.

### Image analysis

Computer assisted image analysis used a Zeiss KS100 system. Digitised microscopic sections are displayed on monitor. The center of the vessels is found by computerised “best fit circle” procedure in normal and circumferentially affected vessels. In non-circumferential lesions further image processing is based on a contour of the unaffected part of the circumference indicated by the observer.

From the center, using a random starting position, 32 radii are drawn every 11.25 degrees (systematic random sampling).

Using interactive procedures, the observer then defines along each displayed radius the position of the endothelium, the lamina elastica interna and externa and the adventitia-fat border. The software then calculates the lumen diameter (r-lu), the radius to various points (r-IEL: radius to Internal Elastic Lamina, etc) and the width of the intima, media and adventitia (Th-int: intimal width, etc) at each location in micrometer. Series of 32 measurements are available for each vessel and are tabulated for further calculations and comparisons.

### Validation of Th-int/r-IEL% as computerised classification parameter for progression of vascular change

In the study of relationships between changes in width among mural constituents related to progression of overall pathology one would prefer to use continuous variables, as this allows for the use of multivariate regression analysis. Thus an objective parameter for overall change is required as an alternative to the classification of pathology given in the earlier section of Materials and Methods. However such a classification of all sections available can be used for simple validation of any proposed parameter.

**Figure 1:**
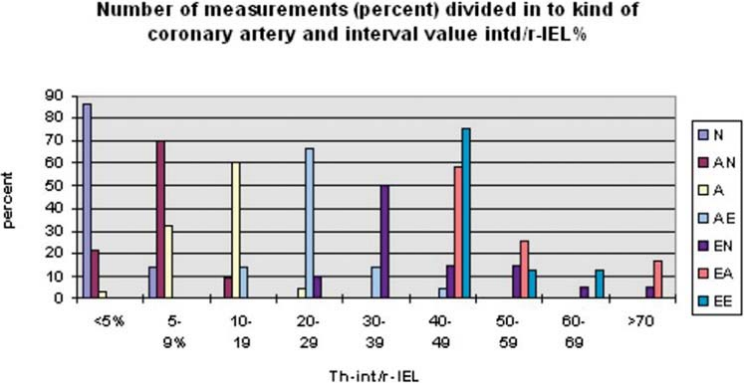
Number of measurements (percent) divided in to histological categories. N: normal coronary artery, AN: possibly abnormal artery-(circumferentially widened intima, width less than media-, A: abnormal artery-artery widened circumferentially, width more than media-, AE: abnormal artery- intima widened non-circumferentially, the remainder of the intima circumference is normal in width-, EN: abnormal artery-extensive, well developed plaque, the remaining circumference is normal in appearance-, EA: abnormal artery: extensive, well developed plaque, the remaining circumference shows pathologic changes-, EE: abnormal artery- circumferential atherosclerotic plaque. Th-int/r-IEL: ratio thickness intima/radius Internal Elastic Lamina.

We calculated the average value for the ratio between the width of the intima (Th-int) and the radius to the Lamina Elastica Interna (r-IEL) for each of the 32 radii of a given vessel (as a %) and compared the values to the results of prior classification. The results are shown in Figure 1. Although there is evident overlap, it is also evident that values for the proposed parameter follow the progression in pathology throughout the whole range of the classification unambiguously. Some of the spread of values must reflect the non-linear relationship between lumen diameter and wall thickness of a contracting or relaxing vessel. In the presence of elongation/shortening concurrent with respect to vessel dilatation/contraction, the relationship between total area on throughsection of intimal tissue and myocellular media (volume sample) is compromised even further.

All samples at postmortem may be taken to have relaxed before and during death followed by contraction/shrinkage with formaldehyde fixation in a comparable manner although not necessarily to an identical degree. In light of the magnitude of changes observed in the initial stages of the study it was felt that an effective signal to noise ratio was maintained. Additionally the parameter corrects for differences in age, body mass, cardiac size and gender related differences in calibre between individuals for the coronary artery under assessment. Therefore we felt that the findings as reflected in Figure 1, allowed for the conclusion that Th-int/r-IEL% is acceptable for use as a continuously variable reference value in the calculations specified below.

### Study of remodelling

For the purpose of studying remodelling, the regression of radius lumen (r-lu) against thickness of the intima (Th-int) was calculated after arranging the datasets for progression of vessel wall pathology (represented by increasing Th-int/r-IEL), using 5% Th-int/r-IEL intervals. This results from the finding that r-lu is linearly related to Th-int with vessels N, AN and A and thus vessels represented by Th-int/r-IEL<20%. By analysing the data sets using 5% Th-int/r-IEL intervals, any gradual changes in pattern can be discerned. Because, in addition to the intima, the media and adventitia may also take part in the remodelling process (either active or passive), the medial and adventitial change of width was studied not only by comparing their mean values for each Th-int/r-IEL interval but also by calculating the percent thickness change, the values of Th-int/t-IEL interval<5% taken as standard.

### Statistical analysis

Using the EXCEL statistical package, regression relationships were calculated using multivariate analysis for all relationships available and resultant data sets assessed for significance. Regression equations and correlation/determination coefficients were calculated and tabled for study. Results for individual groups as defined above were additionally compared using ANOVA statistical analysis package for probability of difference between groups of observations using one-sided, uni-directional comparisons.

## Results

### Validation of indiscriminate use of measurement values coronary arteries N, AN and A and segments of arteries E rated as such

The entire data set consists of measurement data from normal coronary arteries (N), circumferentially enlarged vessels (AN, A) and data from the segments of eccentrically enlarged arteries. The latter, depending on whether the artery was rated as AE, EN, EA, EE, can vary between vessel walls classified as histological N and EE.

As is shown in Figure 1, 100% of the vessels of type N and AN are represented within the area of the graph defined by Th-int/r-IEL<20%. Even for vessels of type A this is the case for 96% of vessels measured.

### Comparison of wall of vessels type N, AN and A to parts of arteries E classified as N, AN or A. These two data sets were prepared using the classification criterium: Th-int/r-IEL<20% values on which the regression of Th-int/r-lu against Th-int/r-IEL was applied

The relative figures Th-int/r-lu and Th-int/r-IEL were used to allow for comparison of differently sized arteries. The regression of the thickness of the media (regression Th-med/r-lu against Th-med/r-IEL) and adventitia (Th-advd/r-lu against Th-adv/r-IEL) was similarly compared. Table 1 shows the results of regression analysis. It is obvious that not only are the slopes of the regression relationships Th-int/r-lu and Th-int/r-IEL almost identical, but that this also is the case for those of Th-med/r-lu against Th-med/r-IEL and those of Th-adv/r-lu against Th-adv/r-IEL. Thus, based on these mathematical findings alone, just as is the case morphologically, these vessels and vessel segments can not be discerned from the segments rated as N, AN and A or arteries and artery segments with Th-int/r-IEL<20%.

**Table 1:**
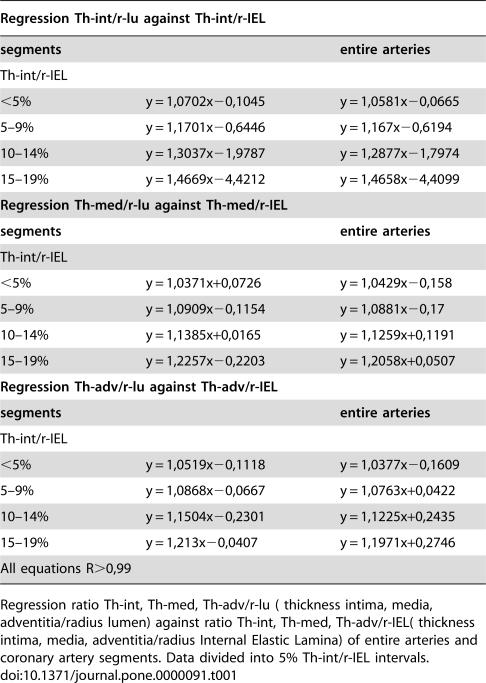
Regression equations of entire arteries and segments.

Regression Th-int/r-lu against Th-int/r-IEL		
**segments**		**entire arteries**
Th-int/r-IEL		
<5%	y = 1,0702x−0,1045	y = 1,0581x−0,0665
5–9%	y = 1,1701x−0,6446	y = 1,167x−0,6194
10–14%	y = 1,3037x−1,9787	y = 1,2877x−1,7974
15–19%	y = 1,4669x−4,4212	y = 1,4658x−4,4099
**Regression Th-med/r-lu against Th-med/r-IEL**		
**segments**		**entire arteries**
Th-int/r-IEL		
<5%	y = 1,0371x+0,0726	y = 1,0429x−0,158
5–9%	y = 1,0909x−0,1154	y = 1,0881x−0,17
10–14%	y = 1,1385x+0,0165	y = 1,1259x+0,1191
15–19%	y = 1,2257x−0,2203	y = 1,2058x+0,0507
**Regression Th-adv/r-lu against Th-adv/r-IEL**		
**segments**		**entire arteries**
Th-int/r-IEL		
<5%	y = 1,0519x−0,1118	y = 1,0377x−0,1609
5–9%	y = 1,0868x−0,0667	y = 1,0763x+0,0422
10–14%	y = 1,1504x−0,2301	y = 1,1225x+0,2435
15–19%	y = 1,213x−0,0407	y = 1,1971x+0,2746
All equations R>0,99		

Regression ratio Th-int, Th-med, Th-adv/r-lu ( thickness intima, media, adventitia/radius lumen) against ratio Th-int, Th-med, Th-adv/r-IEL( thickness intima, media, adventitia/radius Internal Elastic Lamina) of entire arteries and coronary artery segments. Data divided into 5% Th-int/r-IEL intervals.

Based on these results, we considered it justifiable to combine all individual measurements and calculated ratios into a single reference data set for subsequent comparative analyses.

### Remodelling proper

The results of regression analysis of r-lumen against Th-int for 5% intervals of Th-int/r-IEL are shown in Table 2.

**Table 2:**
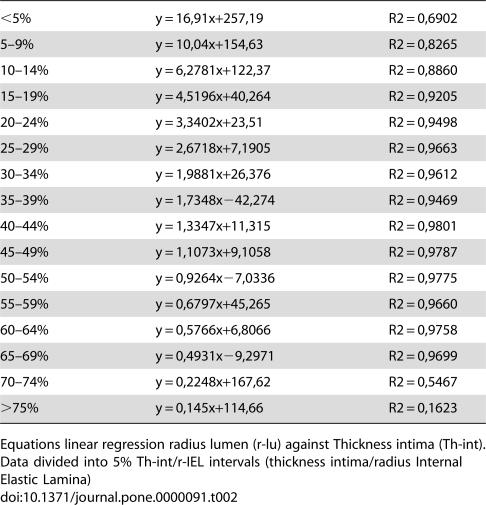
Linear regression radius lumen against thickness intima.

<5%	y = 16,91x+257,19	R2 = 0,6902
5–9%	y = 10,04x+154,63	R2 = 0,8265
10–14%	y = 6,2781x+122,37	R2 = 0,8860
15–19%	y = 4,5196x+40,264	R2 = 0,9205
20–24%	y = 3,3402x+23,51	R2 = 0,9498
25–29%	y = 2,6718x+7,1905	R2 = 0,9663
30–34%	y = 1,9881x+26,376	R2 = 0,9612
35–39%	y = 1,7348x−42,274	R2 = 0,9469
40–44%	y = 1,3347x+11,315	R2 = 0,9801
45–49%	y = 1,1073x+9,1058	R2 = 0,9787
50–54%	y = 0,9264x−7,0336	R2 = 0,9775
55–59%	y = 0,6797x+45,265	R2 = 0,9660
60–64%	y = 0,5766x+6,8066	R2 = 0,9758
65–69%	y = 0,4931x−9,2971	R2 = 0,9699
70–74%	y = 0,2248x+167,62	R2 = 0,5467
>75%	y = 0,145x+114,66	R2 = 0,1623

Equations linear regression radius lumen (r-lu) against Thickness intima (Th-int). Data divided into 5% Th-int/r-IEL intervals (thickness intima/radius Internal Elastic Lamina)

In view of the regression equations, positive remodelling (r-lumen increases more than Th-int) exists for the range Th-int/r-IEL<50% (direction coefficient>1) and negative remodelling for Th-int/r-IEL>50%.

In summary, the distribution of values obtained from pathological vessels, when related to the values for the regression slope (Table 2) shows 3 different relationships.

Firstly, high positive values for the regression slope are found for the segment of values with Th-int/r-IEL up to 20%. There is a subsequent plateau where change, at least for the intimal layer, in wall thickness continues although vessels no longer show changes in lumen diameter (20–50%), followed by a third segment where the regression analysis shows negative slopes with coefficient values<1. In this third phase an effective reduction of lumen diameter is found.

At the end of the first two phases, positive remodelling as has been defined previously, is completed. In the third and assumedly final phase of change, decreasing lumen diameter is associated with disproportional increase in intimal width in a fundamentally altered manner. Intimal width increase in this phase occurs progressively “at the expense” of lumen. This coincides with the phase of negative remodelling as previously defined.

Even though we classified actual vessels studied into classes at 5% intervals, the relationship with observed intimal thickness is remarkable for its gradual change, suggestive of a continuous process.

**Figure 2:**
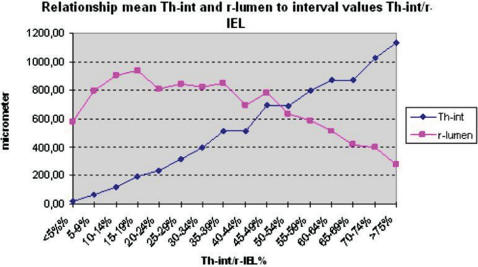
Line diagram relation mean radius lumen (r-lu) and thickness intima (Th-int) for 5% Th-int/r-IEL (thickness intima/radius Internal Elastic Lamina) intervals.

**Figure 3:**
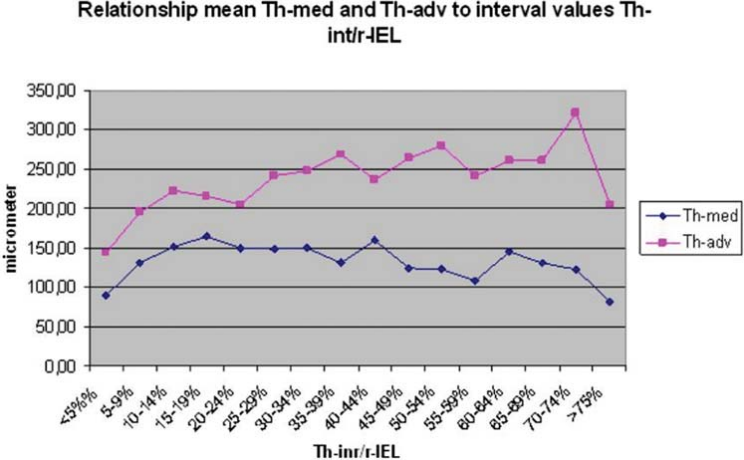
Line diagram relation mean thickness media (Th-med) and thickness adventitia (Th-adv) For 5% Th-int/r-IEL (thickness intima/radius Internal Elastic Lamina) intervals.

**Figure 4:**
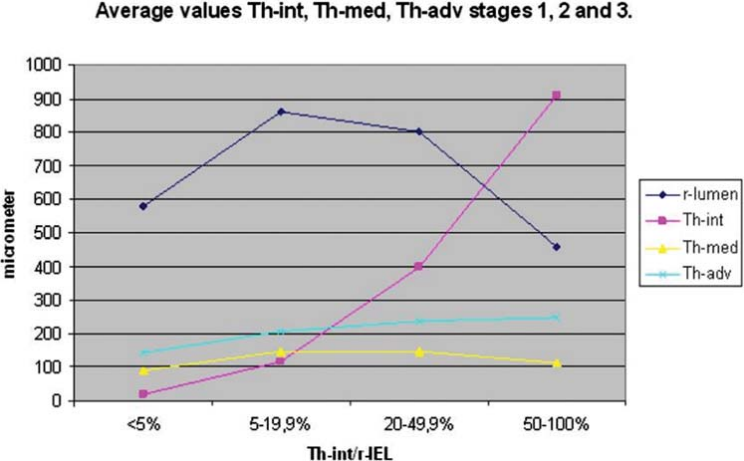
Line diagram mean thickness- intima (Th-int), -media (Th-med), -adventitia (Th-adv) For Th-int/r-IEL (thickness intima/radius Internal Elastic Lamina) reference and stages.

The results of medial and adventitial assessment are presented in Figures 2 and 3. The underlying data are presented in Table 3. Figure 4, while Table 5 present the results of averages calculated for mural components related to succesive stages of the process. From these results it is evident that the three phases recognised for intimal changes may in fact well be present equally in the media and adventitia. The change from phase 1 into 2 is readily recognised to occur in a comparable manner around the 20% demarcation. The subsequent plateau for intima is mirrored in a similar stability of results for media with some continued increase for the values for adventitia. The third phase is similarly present for the media, again commencing around the 50% demarcation. Within the media, based on the data as presented in Table 5 a subdivision may be recognised in phase 3. Here, values for medial width as compared to those of the reference set for vessels with comparable diameters, fall below 100% at a statistically significant level. Re-assessment of the original microscopical slides reveals this change to coincide with the beginning of expansion of lesions originally limited to the intima into the media in a destructive manner. This change is clearly illustrated in Figure 4 and Table 5.

**Table 3:**
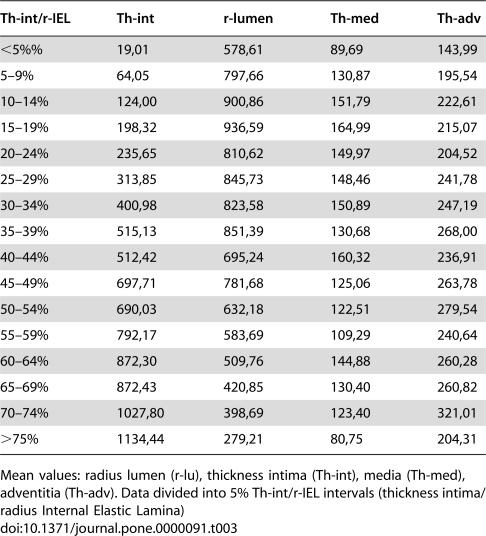
Descriptive statistics.

Th-int/r-IEL	Th-int	r-lumen	Th-med	Th-adv
<5%%	19,01	578,61	89,69	143,99
5–9%	64,05	797,66	130,87	195,54
10–14%	124,00	900,86	151,79	222,61
15–19%	198,32	936,59	164,99	215,07
20–24%	235,65	810,62	149,97	204,52
25–29%	313,85	845,73	148,46	241,78
30–34%	400,98	823,58	150,89	247,19
35–39%	515,13	851,39	130,68	268,00
40–44%	512,42	695,24	160,32	236,91
45–49%	697,71	781,68	125,06	263,78
50–54%	690,03	632,18	122,51	279,54
55–59%	792,17	583,69	109,29	240,64
60–64%	872,30	509,76	144,88	260,28
65–69%	872,43	420,85	130,40	260,82
70–74%	1027,80	398,69	123,40	321,01
>75%	1134,44	279,21	80,75	204,31

Mean values: radius lumen (r-lu), thickness intima (Th-int), media (Th-med), adventitia (Th-adv). Data divided into 5% Th-int/r-IEL intervals (thickness intima/radius Internal Elastic Lamina)

**Table 4:**
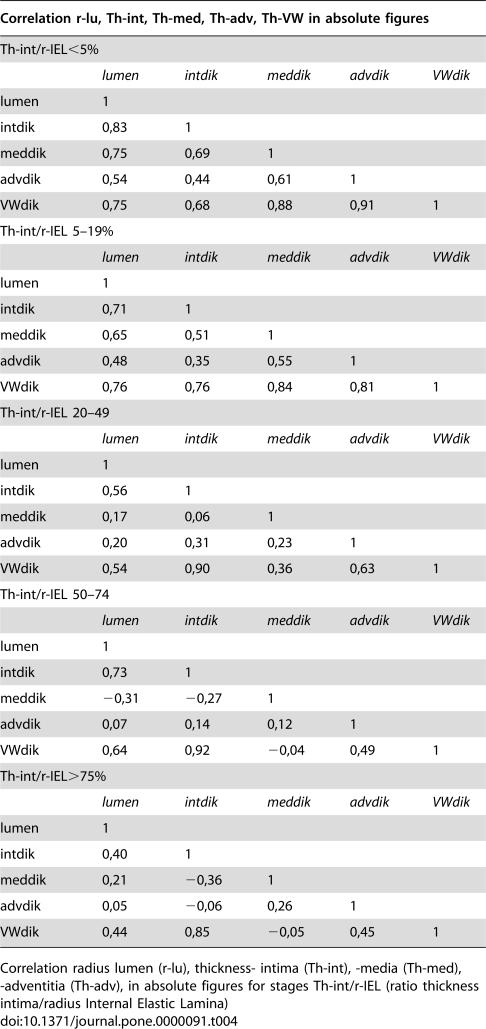
Correlations

Correlation r-lu, Th-int, Th-med, Th-adv, Th-VW in absolute figures					
Th-int/r-IEL<5%					
	*lumen*	*intdik*	*meddik*	*advdik*	*VWdik*
lumen	1				
intdik	0,83	1			
meddik	0,75	0,69	1		
advdik	0,54	0,44	0,61	1	
VWdik	0,75	0,68	0,88	0,91	1
Th-int/r-IEL 5–19%					
	*lumen*	*intdik*	*meddik*	*advdik*	*VWdik*
lumen	1				
intdik	0,71	1			
meddik	0,65	0,51	1		
advdik	0,48	0,35	0,55	1	
VWdik	0,76	0,76	0,84	0,81	1
Th-int/r-IEL 20–49					
	*lumen*	*intdik*	*meddik*	*advdik*	*VWdik*
lumen	1				
intdik	0,56	1			
meddik	0,17	0,06	1		
advdik	0,20	0,31	0,23	1	
VWdik	0,54	0,90	0,36	0,63	1
Th-int/r-IEL 50–74					
	*lumen*	*intdik*	*meddik*	*advdik*	*VWdik*
lumen	1				
intdik	0,73	1			
meddik	−0,31	−0,27	1		
advdik	0,07	0,14	0,12	1	
VWdik	0,64	0,92	−0,04	0,49	1
Th-int/r-IEL>75%					
	*lumen*	*intdik*	*meddik*	*advdik*	*VWdik*
lumen	1				
intdik	0,40	1			
meddik	0,21	−0,36	1		
advdik	0,05	−0,06	0,26	1	
VWdik	0,44	0,85	−0,05	0,45	1

Correlation radius lumen (r-lu), thickness- intima (Th-int), -media (Th-med), -adventitia (Th-adv), in absolute figures for stages Th-int/r-IEL (ratio thickness intima/radius Internal Elastic Lamina)

**Table 5:**
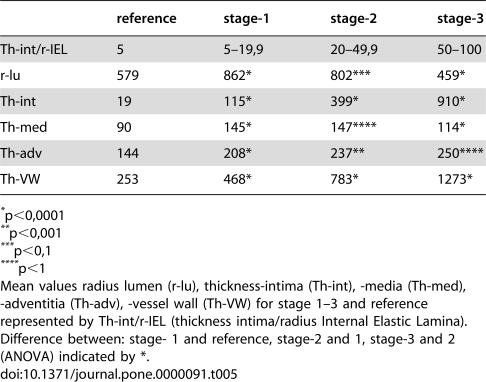
Mean values stage 1, 2, 3 and reference.

	reference	stage-1	stage-2	stage-3
Th-int/r-IEL	5	5–19,9	20–49,9	50–100
r-lu	579	862*	802***	459*
Th-int	19	115*	399*	910*
Th-med	90	145*	147****	114*
Th-adv	144	208*	237**	250****
Th-VW	253	468*	783*	1273*

<?ENTCHAR ast?> p<0,0001

<?ENTCHAR ast?><?ENTCHAR ast?> p<0,001

<?ENTCHAR ast?><?ENTCHAR ast?><?ENTCHAR ast?> p<0,1

<?ENTCHAR ast?><?ENTCHAR ast?><?ENTCHAR ast?><?ENTCHAR ast?> p<1

Mean values radius lumen (r-lu), thickness-intima (Th-int), -media (Th-med), -adventitia (Th-adv), -vessel wall (Th-VW) for stage 1–3 and reference represented by Th-int/r-IEL (thickness intima/radius Internal Elastic Lamina).

Difference between: stage- 1 and reference, stage-2 and 1, stage-3 and 2 (ANOVA) indicated by *.

### Results of ANOVA analysis

ANOVA analysis confirmed the internal coherence of distribution of parameters within the defined reference group. Values for the parameter distribution (Th-int/r-IEL) defining groups, differed between groups at high significance (p<4.72e-14), as would be predicted but confirming appropriateness of defining criterion.

**Figure 5:**
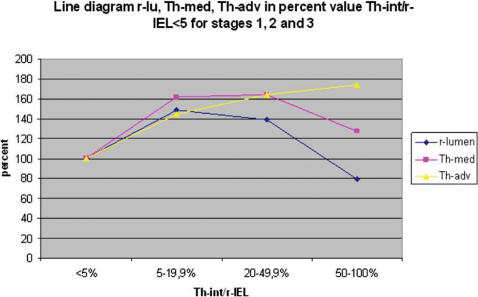
Line diagram radius lumen (r-lu), thickness media (Th-med) and thickness adventitia (Th-adv) in percent of reference values Th-int/r-IEL<5 (100%) for stages Th-int/r-IEL (thickness intima/radius Internal Elastic Lamina).

Subsequent analysis of changes in study parameters (reflecting medial and adventitial changes) associated with grouping of vessels studied, resulted in equally low probabilities of non-difference (p<0.001). From stage 2 to stage 3 , a more limited change is seen with a slightly larger distribution, resulting in a p value of non-difference of approximately 4%. As can be seen in Table 5, Figure 4 and 5, the process differs between media and adventitia, with the media reducing in thickness both in absolute terms, whereas the adventitia continues to increase in thickness, both in absolute and in proportional terms.

## Discussion

Not only the intima but also the media and adventitia will play a role in the remodelling process, after all the three layers, not only anatomically but also functionally/physiologically compose the vessel wall. The mean figures of r-lumen, Th-int, Th-med and Th-adv for various Th-int/r-IEL intervals, are presented in Table 3 and Figures 2 and 3. It is obvious that Th-med and Th-adv increase with Th-int/r-IEL<20% and, considering the correlation coefficients (Table 4), are proportionate to r-lu and Th-int, although with Th-med this relationship is statistically stronger than with Th-adv. With Th-int/r-IEL>20% the curves of Th-med and Th-adv differ, Th-med remains more or less constant in its relationship during the phase of Th-int/r-IEL 20–50% and decreases when Th-int/r-IEL>50% while Th-adv continues to increase when the ratio for Th-int/r-IEL has reached 20% until Th-int/r-IEL has reached values>75%.

This phenomenon is also displayed in Table 4, Th-med is positively related to r-lu and Th-int up to the point were Th-int/r-IEL has reached a value of 50%. After this the value becomes inversely related to r-lu Th-int/r-IEL until the value of approximately 50–75% when it again becomes positively related with Th-int/r-IEL after the value of>75%.

Th-adv, on the contrary, is positive related to r-lumen and Th-int of all Th-int/r-IEL intervals until Th-int/r-IEL 75%. With Th-int/r-IEL>75% Th-adv also decreases.

The conclusions mentioned above (Figure 5) are also expressed in the percent change of the mean values of r-lu, Th-int, Th-med, Th-adv and Th-VW for each Th-int/r-IEL interval, the mean value of Th-int/r-IEL interval<5% taken as standard (100%). All this support the notion remodelling to be a three stage process dependent on Th-int and Th-med and in a lesser way Th-adv.
